# Normative data on spontaneous stride velocity, stride length, and walking activity in a non-controlled environment

**DOI:** 10.1186/s13023-021-01956-5

**Published:** 2021-07-19

**Authors:** Margaux Poleur, Ana Ulinici, Aurore Daron, Olivier Schneider, Fabian Dal Farra, Marie Demonceau, Mélanie Annoussamy, David Vissière, Damien Eggenspieler, Laurent Servais

**Affiliations:** 1 Centre de Référence des Maladies Neuromusculaires, Centre Hospitalier Régional de la Citadelle, Boulevard du 12eme de Ligne 1, 4000 Liège, Belgium; 2 Sysnav, Vernon, France; 3 Department of Paediatrics, MDUK Oxford Neuromuscular Centre, University of Oxford, Oxford, UK

**Keywords:** Accelerometer, Home monitoring, Healthy volunteers, Stride length, Stride velocity

## Abstract

**Background:**

Normative data are necessary for validation of new outcome measures. Recently, the 95th centile of stride speed was qualified by the European Medicines Agency as a valid secondary outcome for clinical trials in subjects with Duchenne muscular dystrophy. This study aims to obtain normative data on spontaneous stride velocity and length in a non-controlled environment and their evolution after 12 months.

**Method:**

Ninety-one healthy volunteers (50 females, 41 males), with a mean age of 16 years and 2 months, were recruited and assessed at baseline and 12 months later. The 4-stair climb, 6-min walk test, 10-m walk test and rise from floor assessments were performed. Stride length, stride velocity, and the distance walked per hour were studied in an everyday setting for one month after each evaluation.

**Results:**

Of the 91 subjects assessed, 82 provided more than 50 h of recordings at baseline; and 73 subjects provided the same at the end of the year. We observed significant positive correlations of the stride length with age and height of participants, and a significant increase of the median stride length in children after the period. In this group, the 95th centile stride velocity was not correlated with age and was stable after one year. All measures but the 10MWT were stable in adults after a one-year period.

**Conclusion:**

This study provides with data on the influence of age, height, and gender on stride velocity and length as well as accounting for natural changes after one year in controls.

## Background

Duchenne muscular dystrophy (DMD) is a severe, rapidly progressive neuromuscular disorder characterized by muscle weakening. It has an estimated incidence of 1 in 5000 males [1]. DMD is caused by out-of-frame mutations in the *dystrophin* gene, which leads to an absence or deficiency of the protein dystrophin and the degeneration of muscles fibres [2]. Therefore, loss of ambulation occurs generally around the age of 12.

The 6-min walk distance test (6MWT) has been used as a primary outcome in most published pivotal phase 2 and 3 trials in ambulant patients with DMD [3–6]. Other outcomes used include the 4-stair climb (4SC) and functional scales such as the North Star Ambulatory Assessments (NSAA) in which participants are rated on their ability to perform standardized motor function tasks [3, 5–10]. In DMD, the low Standardized Response Mean of these different outcome measures, which illustrates the power of these measures to demonstrate a change over a certain period of time, meant that pivotal trials required over 100 patients per group and trial durations of 18 months and 2 years (NCT02851797 and NCT02500381, respectively). To accelerate clinical development and investigate in parallel several approaches without being limited by the number of patients available, it is crucial to validate more powerful outcome measures. This is the case for DMD and for numerous other rare diseases, within or outside the neuromuscular field.

The last decade has seen an increase in the availability of wearable technology for continuous monitoring of health and wellbeing [11]; for example, wearable devices that can be used to assess ambulation range from crude step counters to sophisticated multisensory systems [12]. Unlike consumer devices, medical devices must demonstrate validated measurement accuracy, sensitivity, and specificity [13]. Wearable devices have the potential to provide a complete view of a patient’s condition over a long time period by band-pass filtering day-to-day variation. Therefore, wearable devices provide a complementary approach with a major advantage over hospital-based assessments, which only provide ‘snapshots’ of a patient’s condition that can be affected by fatigue, illness, or lack of motivation.

In this context, Sysnav, a medium size private company, along with academic partners specifically developed a CE-marked class 1 wearable medical device, called Actimyo®, that records passively, in a precise and sensitive way, upper and lower limb movements in everyday life [14, 15]. From the capture of any single movement, several outcomes may be extracted. In ambulant patients, the identification and quantification of every individual stride allows for the calculation of the distribution of stride length and stride speed as well as for the analyses of different centiles such as the 95th centile stride velocity (SV95C). These variables are measured in a home-based environment over a 180-h period and are reliable and highly sensitive to changes in ambulant patients with DMD [16].

Recently, the EMA qualified SV95C as a valid secondary endpoint in clinical trials on ambulant patients with DMD [17]. Additional data are needed to qualify the measure as a primary endpoint [16]. Wearable technology is or has been used in clinical trials of therapies for spinal muscular atrophy [18, 19], facioscapulohumeral muscular dystrophy (NCT02579239, NCT04004000), limb girdle muscular dystrophy type 2E (NCT02579239), centronuclear myopathy (NCT02057705), and Angelman syndrome (NCT04259281).

To properly interpret the longitudinal evolution of the SV95C over time in patient populations, it is necessary to understand the longitudinal evolution in subjects without muscle conditions within the same age range, in particular between 5 and 18 years old, which is the age range mostly targeted in clinical trials of ambulant DMD patients and during which growth or maturational factors may significantly interfere with measures [20, 21]. Therefore, we conducted a longitudinal study in ambulant healthy subjects between 5 and 85 years of age to evaluate the changes in SV95C, as measured with the Actimyo® device, after a 12-months follow-up.

## Method

### Study design

We designed a monocentric academic study that was conducted in the Reference Centre for Neuromuscular Disease in Liège, Belgium between July 2017 and September 2019 with grant funding from Action Duchenne. The protocol was approved by the local ethics committee in Liège (N/Ref:1646). Before inclusion, all participants or parents or legal guardians provided written informed consent for participation and publication.

### Participants

The initial protocols planned to include a maximum of 130 healthy subjects to gather a distribution of about 5–10 subjects par age year in children and a group of 30 adults. However, due to difficulties in recruiting controls—mainly in the adult age group—91 healthy subjects above the age of 5 years were assessed at baseline and 84 were assessed after 12 months. The exclusion criteria were occurrence of surgery or recent trauma in the upper or lower limbs within six months prior to the first visit; participating in sport at a high (national) level; pregnant or breastfeeding women; occurrence of muscular, neurological, infectious, acute, or chronic inflammatory disease within three weeks of inclusion date; and occurrence of orthopaedic, neuromuscular, or neurological disease with an impact on the quality of the walk.

Demographic data as well as medical and surgical histories were obtained during the baseline visit. A full physical examination, including weight, height, and vital signs, was performed at the first visit and at the 12-month visit. The subjects performed the 6MWT while wearing the sensors at baseline and at 12 months. They also performed the 4SC, rise from floor (RFF), and 10-m walk test (10MW). Assessment of dynamometric grip and pinch strength were evaluated with the Myogrio and Myopinch, as previously described [22]. All tests were conducted by trained and certified physiotherapists from the neuromuscular centre.

### Timed tests

The subjects performed the 6MWT according to the modified ATS guidelines [23]. For RFF, trained examiners recorded using a stopwatch the time taken by the patient to complete the task of rising from supine to standing as fast as possible. For the 4SC, the subject had to climb a four-step flight of stairs as fast as possible. For the 10MW, the subject had to walk or run for ten meters in a straight line. We recorded the time taken to complete the task as fast as possible. In order to ascertain that the patient’s best performance is captured, each of these tests were performed three times with a 1-min rest between each trial and we kept the test performed at the highest speed.

### Movement monitoring

The ActiMyo® device (Sysnav, Vernon, France) was worn for 1 month at baseline and at 12 months. The two sensors, each containing a magneto-inertial sensor that records the linear acceleration, the angular velocity and the magnetic field of the movement in all directions, were fixed to the dominant wrist and ankle. According to the EMA, for the purposes of qualification, a recording is considered as valid if it includes at least 50 h of recording; optimally it should include 180 h of recording [16]. We studied three different variables: stride length, stride velocity, and the number of meters walked per hour. Stride length and stride velocity were studied as the medians (SL50C and SV50C) or the 95th centiles (SL95C or SV95C).

Upper limb measurement is still a work in progress and will be presented elsewhere.

### Statistical analysis

For analysis, data were excluded from subjects who recorded less than 50 h; 9 subjects at baseline and 18 subjects at follow-up, respectively. Analysis was first performed on the whole sample and then we analysed children (5 to 17 years) and adults (18 years and older) separately. Then within these groups, we analysed male and female subgroups. We used a series of Mann–Whitney U tests to compare subgroups. We then performed correlation analyses using Spearman’s rank correlation coefficient based on accelerometer measures, time test performances, age, and height. Finally, we use the Wilcoxon test to assess the evolution of each measure after 12 months. For compliance analysis, we used the 50-h and 180-h thresholds as these two thresholds were defined in the EMA qualification document as acceptable and optimal, respectively (16). All analyses were performed using IBM’s SPSS Statistics software. The limit of statistical significance was set to 0.05.

## Results

### Population

**Fig. 1:**
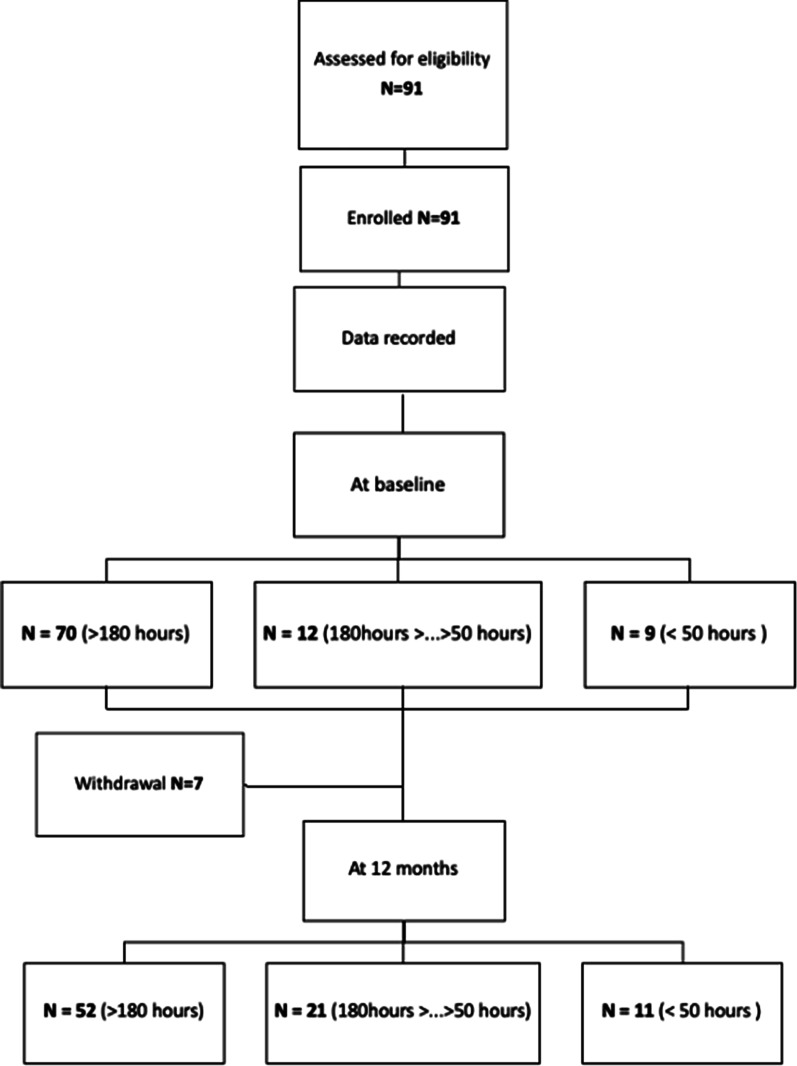
Flow-chart displaying the compliance and inclusion of subjects. Withdrawals resulted from loss of follow up (5 subjects) and withdrawal by the subject (2 subjects)

We included 91 healthy individuals (41 males (45.1%) and 50 females (54.9%) aged between 6 and 85 years, with a mean age of 16 years 2 months. Seven participants withdrew between the baseline and the follow-up visit for personal reasons. A flow chart of subject participation is shown in Fig. 1. The demographic and clinical characteristics of participants whose baseline data were analysed, as well as ActiMyo® variables, are provided in Table 1.

**Table 1:** Demographic and clinical characteristics of participants who recorded more than 50 h

Characteristics				
	All	Adult	Children	
			All	Male
Sample size	82	17	65	31
Age (years)	16.9 (16.0)	43 (18.9)	10.1 (2.8)	10.6 (2.9)
	[6.0–84.3]	[18.0–84.3]	[6.0–17.6]	[6.1–17.6]
Sex: number of female (%)	46 (56.0%)	12 (70,6%)	34 (52.3%)	0 (0%)
Height (cm)	144.0 (18.3)	167.5 (10.6)	137.8 (14.6)	142.5 (15.1)
	[114.0–195.0]	[153.2–195.0]	[114.0–173.3]	[118.0–174.3]
BMI (kg/m^2^)	18.8 (4.5)	25.7 (3.6)	17.0 (2.5)	17.2 (2.4)
	[13.2–32.8]	[20.1–32.8]	[13.2–26.1]	[13.9–23.7]
6MWT (m)	594.7 (71.2)	551.5 (82.0)	606.0 (64.1)	618.2 (61.6)
	[436.0–761.0]	[436.0–716.0]	[464.0–761.0]	[496.0–761.0]
4SC (s)	1.4 (0.3)	1.6 (0.4)	1.3 (0.3)	1.3 (0.3)
	[0.9–2.3]	[1.1–2.3]	[0.9–2.3]	[1–2.3]
RFF (s)	1.8 (0.6)	2.7 (0.9)	1.6 (0.2)	1.57 (0.3)
	[1.0–4.9]	[1.2–4.9]	[1.0–2.2]	[1.0–2.2]
10WT (m)	4.8 (0.8)	5.1 (0.8)	4.7 (0.7)	4.5 (3.5)
	[3.1–7.3]	[4.1–7.3]	[3.1–7.2]	[3.5–5.7]
Distance/hour (m/h)	265.2 (87.2)	246.4 (89.5)	270.2 (86.5)	294.8 (88.9)
	[39.9–484.4]	[119.6–433.0]	[39.9–484.4]	[39.9–484.4]
SV50C (m/s)	1.0 (0.1)	1.1 (0.1)	1.0 (0.1)	1.0 (0.1)
	[0.7–1.3]	[0.7–1.3]	[0.9–1.3]	[0.9–1.3]
SV95C (m/s)	2.4 (0.6)	1.6 (0.3)	2.6 (0.4)	2.6 (0.4)
	[1.1–3.6]	[1.1–2.3]	[1.6–3.6]	[1.7–3.2]
SL50C (m)	1.1 (0.1)	1.2 (0.1)	1.0 (0.1)	1.1 (0.1)
	[0.8–1.5]	[0.8–1.5]	[0.8–1.4]	[0.8–1.4]
SL95C (m)	1.7 (0.2)	1.5 (0.2)	1.7 (0.2)	1.7 (0.2)
	[1.1–2.3]	[1.1–1.8]	[1.4–2.3]	[1.4–2.1]

Data are expressed in mean (SD) [Min–Max]. *BMI* body mass index, *6MWT* 6-min walk distance, *4SC* 4-stair climb; *RFF* rise from floor; *10MW* 10-m walk test; *SV50C* 50th centile stride velocity; *SV95C* 95th centile stride velocity; *SL50C* 50th centile stride length; *SL95C* 95th centile stride length

Statistical analysis was performed on data from the 82 participants who achieved more than 50 h of recording at baseline. For further analysis, healthy subjects were distributed into two age subgroups: those 5 to 17 years of age and those aged 18 years and older. The first subgroup was composed of 65 participants, 34 of whom were females (52.3%). The second group was composed of 17 participants, of whom 12 were females (70.6%).

The two age groups differed from each other in most measures including height, 6MWD, 4SC, RFF, and stride characteristics (SV50C, SV95C, SL50C and SL95C). Younger subjects were shorter (*p* < 0.001) but had a higher 6MWD (*p* = 0.011) than adults. The younger group also performed better at 4SC (*p* = 0.001) and RFF (*p* < 0.001). The groups did not differ significantly in 10MW performance (*p* = 0.079). Regarding measurements obtained with the magneto-inertial device, the groups did not differ significantly in the distance walked per hour (*p* = 0.215). SV50C tended to be higher in the adult group (*p* = 0.047), whereas SV95C was higher in children (*p* = 0.001). We found a similar pattern in stride length measures: the median (SL50C) was higher in adults (*p* = 0.001) but the 95th centile (SL95C) was higher in children (*p* = 0.001). The ratio between the SV95C and the SV50C was three times higher in younger subjects than in adults, and the ratio between SL95C and SL50C was twice higher in younger subjects, which seems to indicate a more stereotyped walking patterns in adults.

### Compliance in wearing the device

Among the 91 healthy controls who wore the wearable device for one month, 90% recorded more than 50 h, and 77% recorded more than 180 h at baseline. During the second period, 86% recorded more than 50 h, and 73% recorded more than 180 h. The main reason reported by subjects who recorded less than 50 h was ‘technical issues’. There was no age or gender effect evident on the recording duration (data not shown).

### Correlations between timed tests, stride parameters and walking activity

Table 2 presents correlation coefficients between stride parameters obtained using the wearable device and clinical measures at baseline in both subgroups. We also present data from the 82 subjects who achieved more than 50 h of recording at baseline. 6MWD was significantly correlated with SV50C, SV95C, and SL95C in the younger group. In the adult group, 6MWD was only correlated with ‘maximum performance’ measures SV95C and SL95C. In the younger population, the impact of age and height was more obvious than in the adult population (Table 2). Among the group aged 5 to 17 years, height was highly correlated with age, 6MWD, 10MW, SV50C, SL50C, and weakly correlated with SV95C and SL95C. In adults, height was only correlated with stride length measures SL50C and SL95C. We found no correlation between recording duration and stride length, stride velocity, or walking activity (distance/h, *p* = 0.323; SV50C, *p* = 0.466; SV95C, *p* = 0.664; SL50C, *p* = 0.723 and SL95C, *p* = 0.798).

**Table 2:** Correlation between accelerometer measures and clinical measures (Spearmen coefficient)

		Age	Height	BMI	6MWT	4SC	RRF	10MW	Distance	SV50C	SV95C	SL50C	SL95C
Age	5–17 y		0.927**	0.667**	0.122	0.184	0.418**	− 0.143	− 0.083*	0.513**	− 0.609**	0.829**	− 0.099*
	≥ 18 y												
Height	5–17 y	0.909**		0.620**	0.157	0.137	0.389**	− 0.149	− 0.135	0.499**	− 0.598**	0.883**	− 0.037
	≥ 18 y	− 0.134											
BMI	5–17 y	0.363**	0.356**		− 0.204	0.057	0.390**	0.065	− 0.091	0.321**	− 0.575**	0.510**	− 0.363**
	≥ 18 y	0.627**	0.189										
6MWD	5–17 y	0.6**	0.538**	− 0.015		− 0.135	− 0.334**	− 0.565**	0.101	0.336**	0.271*	0.212	0.465**
	≥ 18 y	− 0.703**	0.061	− 0.559*									
4SC	5–17 y	− 0.142	− 0.163	− 0.303*	− 0.033		0.607**	0.256*	− 0.285**	− 0.077	− 0.3**	0.057	− 0.224*
	≥ 18 y	0.14	− 0.288	0.181	0.054								
RFF	5–17 y	− 0.011	0.028	− 0.008	− 0.183	0.515**		0.181	− 0.298**	− 0.01	− 0.631**	0.289**	− 0.494**
	≥ 18 y	0.447	0.01	0.432	− 0.409	0.63**							
10MW	5–17 y	− 0.479**	− 0.407**	− 0.113	− 0.595**	0.155	0.089		− 0.362**	− 0.377**	− 0.203	− 0.276*	− 0.328**
	≥ 18 y	0.434	− 0.326	0.225	− 0.373	0.415	0.362						
Distance	5–17 y	0.03	− 0.036	0.013	0.11	− 0.232	− 0.263*	− 0.426**		0.455**	0.389**	0.081	0.289**
	≥ 18 y	0.115	0.108	0.174	− 0.078	− 0.43	− 0.241	− 0.201					
SV50C	5–17 y	0.621**	0.552**	0.292*	0.454**	− 0.219	− 0.222	− 0.477**	0.451**		− 0.053	0.74**	0.293**
	≥ 18 y	− 0.206	0.116	− 0.113	0.444	− 0.087	− 0.018	− 0.331	0.691**				
SV95C	5–17 y	− 0.231	− 0.269*	− 0.207	0.034	− 0.117	− 0.418**	− 0.09	0.437**	0.114		− 0.414**	0.734**
	≥ 18 y	− 0.738*	0.254	− 0.397	0.858**	0.05	− 0.385	− 0.306	0.157	0.542*			
SL50C	5–17 y	0.869**	0.876**	0.285	0.518**	− 0.163	− 0.1	− 0.493**	0.148	0.786**	− 0.098		0.16
	≥ 18 y	− 0.091	0.678**	− 0.145	0.103	− 0.27	0	− 0.331	0.659**	0.684**	0.417		
SL95C	5–17 y	0.312*	0.274*	− 0.142	0.374**	− 0.144	− 0.405**	− 0.298*	0.281*	0.418**	0.728**	0.455**	
	≥ 18 y	− 0.561*	0.668**	− 0.093	0.485*	− 0.187	− 0.256	− 0.319	0.252	0.449	0.748**	0.721**	

Significance level *p* ≤ 0.01**, *p* ≤ 0.05*. Upper part: correlation on the whole sample. Lower part: correlation on the two age groups. *BMI* body mass index, *6MWT* 6-min walk distance, *4SC* 4-stair climb; *RFF* rise from floor; *10MW* 10-m walk test; *SV50C* 50th centile stride velocity; *SV95C* 95th centile stride velocity; *SL50C* 50th centile stride length; *SL95C* 95th centile stride length

### Gender effect

In the adult population, males showed statistically significantly higher SL50C (*p* = 0.009) and SL95C (*p* = 0.001), probably because they are on average taller than women in the cohort (*p* = 0.048). There was no other difference between males and females in the adult group. In contrast, in the younger population, male subjects were taller than female subjects, but we did not detect significant differences in any qualitative real-life walking parameter between the two subgroups.

### Longitudinal data

**Fig. 2:**
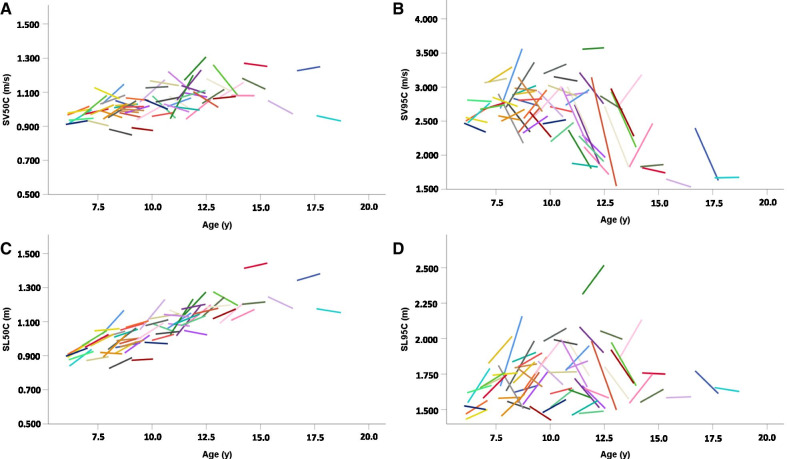
One year evolution of SV50C (**A**), SV95C (**B**), SL50C (**C**) and SL95C (**D**) in subjects aged 5 to 17 years. *SV50C* 50th centile stride velocity; *SV95C* 95th centile stride velocity; *SL50C* 50th centile stride length; *SL95C* 95th centile stride length

Changes after 12 months are presented in Table 3 and evolution of the measures obtained with the magneto-inertial device in children are displayed in Fig. 2. We present data on the 73 subjects who achieved more than 50 h of recording both at baseline and on follow-up visits. Given the effects of growth, we investigated two subgroups separately. In the younger group, we observed a significant increase in functional measures (6MWD, 4SC, 10MW) after one year. Of the measures obtained with the magneto-inertial device, only SL50C increased after one year. We did not observe a significant change after a year in adults in stride length, stride velocity, distance walked, 6MWD, or RFF with the exception of a slight improvement in the 10MW performance.

**Table 3:** Evolution of measures after one year

Subgroup	5–17 years old		Over 18 years old	
Characteristics	Mean (SD)	Sig	Mean (SD)	Sig
Δ6MWD (m)	33.6 (50.302)	*p* = 0.000**	14.87 (46.649)	*p* = 0.191
Δ4SC (s)	− 0.11 (0.288)	*p* = 0.004**	− 0.02 (0.463)	*p* = 0.875
ΔRFF (s)	− 0.07 (0.336)	*p* = 0.293	− 0.24 (0.736)	*p* = 0.239
Δ10MW (s)	− 0.76 (0.77)	*p* = 0.000**	− 0.52 (0.632)	*p* = 0.011*
ΔDistance (m/h)	− 17.23 (76.17)	*p* = 0.059	9.38 (67.15)	*p* = 0.609
ΔSV50C (m/s)	0.01 (0.078)	*p* = 0.359	0.01 (0.154)	*p* = 0.691
ΔSV95C (m/s)	− 0.12 (0.447)	*p* = 0.112	0.12 (0.389)	*p* = 0.955
ΔSL50C (m)	0.05 (0.06)	*p* = 0.000**	0.01 (0.095)	*p* = 0.427
ΔSL95C (m)	0,02 (0.184)	*p* = 0.241	0,04 (0.133)	*p* = 0.691

Time effect was assessed by a rank Wilcoxon test for single sample (null hypothesis: median = 0). Significance level *p* ≤ 0.01**, *p* ≤ 0.05*. 6MWT: 6-min walk distance, 4SC: 4-stair climb; RFF: rise from floor; 10MW: 10-m walk test; SV50C: 50th centile stride velocity; SV95C: 95th centile stride velocity; SL50C: 50th centile stride length; SL95C: 95th centile stride length

## Discussion

As a result of this study, we provide normative data for spontaneous stride velocity, stride length and walking distance per hour obtained passively with a wearable device during a one-month recording period in a non-controlled setting and the one-year evolution thereafter. Most previous studies using wearable devices provide us with quantitative parameters [24]. Qualitative gait analysis as stride length and velocity were mostly obtained based on evaluations inside clinical facilities [25, 26]. Recently, several efforts were made to collect, in a home-based setting, qualitative data from subjects with various diseases and control populations [18, 19, 22, 27]. However, to the best of our knowledge, we found no other published data regarding normative longitudinal evaluations of spontaneous stride velocity, stride length, and walking activity in an everyday setting.

As suggested in previous findings [28], acquiring normative data was feasible. The overall compliance with device use was good. More than 90% of subjects wore the device more than the minimum requirement of 50 h, which indicates that wearing devices at the ankle and at the wrist for one month was acceptable for most participants. The main reason given for data collection below the 50-h threshold was ‘technical issues’, and compliance was not influenced by gender or age. In addition, the recording duration did not influence the variables that were studied.

As expected, results of timed tests were growth dependent [22]. We also observed the influence of height (in adults) and height and growth (in children) on stride length. These results support previous findings [29, 30]. Height did not influence SV95C, for which only a very weak inverse correlation was found with height in the adult subgroup only. In children, all correlations between age, height and stride length or speed were much weaker with maximum performance as measured by the 95th centile than with median values. Independence of growth is an advantage in clinical trials that are conducted in children over 1 or 2 years, especially in DMD where growth is altered by steroid use.

In comparison to ambulant DMD patients of the same age, healthy children present a 67.3% higher SV95C [17]. The difference for other variables was not as extreme, and there is some overlap between DMD and control participants (SL50C 31.39%, SL95C 52.41%, SV50C 25.72%, distance walked/hour 63.13%) [16]. SV95C was stable after one year in controls but significantly and constantly decreases in subjects with DMD. In DMD patients, there is a significant correlation between SV95C and all timed tests [17]. We did not find such correlations in controls, which is probably related to the fact that weakness constitutes a common limiting factor on 6MWD and SV95C in patients with DMD, strengthening the correlation in this group.

Gathering normative data is crucial in the validation process of an outcome measure, as it allows identification of potentially confounding variables such as age, height, or gender. For a specific outcome, the normative data can either show a strong ceiling effect, as is the case for most of normative scales such as CHOP Intend, NSAA, and Motor Function Measure [31–34], or increases with age, as is the case for strength [21, 35] and the 6MWD [20].

The limitations of this study reside in the small number of controls per subgroup (age and gender), principally related to the limited availability of recording devices. Given the number of subjects, we did not study the seasonal influence on the parameters. It is likely that a seasonal analysis would need to take weather conditions into account, as they are more likely to influence walking behaviour than does the time of year, and would require continuous recording over one year, which could be burdensome in controls. Another limitation is that the BMI evolution of participants tends toward a more gradual increase than those of age-matched DMD, mainly after 8 years of age [36]. However, this is not a major issue since there is no correlation between BMI and SV95C in both the adult and children subgroups. During the study, participants wore devices on both wrist and ankle. The analysis of upper limb movement in control patients is complex, since there is an interference with movement produced during the ambulation. The results will therefore be presented elsewhere, with comparison to the upper limb measures in ambulant and non-ambulant DMD patients.

## Conclusion

This study provides evidence that normative data regarding stride length and velocity in a home-based environment with wearable sensors is feasible. These normative data will allow us to express a patient’s data as a percentage of predicted value for age or height and to identify those variables that have important confounding factors. There is still a large amount of work to be done to enable analysis of ambulation and upper limb function in various diseases using medical device for real-life mobility measurement. Future research should focus on exploring new fields of neurological disease and the creation of algorithms to study upper limb function, falls, or ataxia.

## Data Availability

The datasets used and/or analysed during the current study are available from the corresponding author on reasonable request.

## Abbreviations

4SC: 4-Stair climb
6MWT: 6-Minute walk test
6MWD: 6-Minute walk distance
10MW: 10-Meter walk test
BMI: Body mass index
DMD: Duchenne muscular dystrophy
EMA: European Medicines Agency
NSAA: North Star Ambulatory Assessments
RFF: Rise from floor
SV95C: 95Th centile stride velocity
SV50C: 50Th centile stride velocity
SL95C: 95Th centile stride length
SL50C: 50Th centile stride length
